# Beyond A Single Study Visit: Lesson Learned from the AD Clinical Trials

**DOI:** 10.1002/alz70859_099570

**Published:** 2025-12-25

**Authors:** Jinglin Zhong, David Li, Paul Delmar, Guoqiao Wang

**Affiliations:** ^1^ Alector, South San Francisco, CA USA; ^2^ Eisai Inc., Nutley, NJ USA; ^3^ F. Hoffmann‐La Roche Ltd, Basel Switzerland; ^4^ Washington University School of Medicine, St Louis, MO USA

## Abstract

**Background:**

Recent clinical trials for Alzheimer’s disease have provided key insights: (i) the treatment effect is relatively proportional across post‐baseline visits; and (ii) consistent effects can be observed across primary and secondary endpoints. These findings prompt rethinking traditional mixed models for repeated measures (MMRM) to better leverage post‐baseline longitudinal data. Traditional MMRM focuses on comparing mean changes from baseline at the last study visit (MMRM‐Last‐Visit). While this method is accepted by regulatory agencies for its minimal constraints on disease progression patterns, it is inefficient as treatment efficacy inference does not fully utilize mean changes at earlier visits. This study aims to propose alternative methods that utilize all or multiple post‐baseline visits to improve statistical efficiency.

**Method:**

Using simulated semi‐real trial data mimicking disease progression observed in recent Alzheimer’s trials (e.g., Clarity‐AD, TRAILBLAZER‐ALZ 2, GRADUATE I&II), we illustrate several models that leverage multiple post‐baseline visits, including: MMRM‐multiple‐visits, proportional MMRM, cubic spline MMRM, and proportional cubic spline MMRM models. Examples from other neurodegenerative diseases will also demonstrate the feasibility of these models. Additionally, global test statistics using multiple endpoints will be explored.

**Result:**

Estimating treatment effects across all or multiple post‐baseline visits can reduce required sample size by 20% or more. The MMRM‐multiple‐visits model is a natural extension of MMRM‐Last‐Visit, requiring no additional assumptions, and can serve as an alternative primary analysis method. Strengths and limitations of the alternative models will be evaluated using semi‐trial data. Additionally, global test statistics incorporating multiple endpoints will be presented for each method.

**Conclusion:**

Disease‐modifying treatments often show early and consistent effects throughout trials. Analytical models that leverage these characteristics can significantly reduce sample size. Future clinical trials could accelerate treatment development by adopting models that utilize multiple visits as the primary analysis while retaining MMRM‐Last‐Visit for sensitivity analysis. Under a disease‐modifying treatment, the estimated disease progression patterns from the primary and sensitivity analysis models are expected to align consistently.

